# Early astrocytosis in autosomal dominant Alzheimer’s disease measured *in vivo* by multi-tracer positron emission tomography

**DOI:** 10.1038/srep16404

**Published:** 2015-11-10

**Authors:** Michael Schöll, Stephen F. Carter, Eric Westman, Elena Rodriguez-Vieitez, Ove Almkvist, Steinunn Thordardottir, Anders Wall, Caroline Graff, Bengt Långström, Agneta Nordberg

**Affiliations:** 1 Department NVS, Center for Alzheimer Research, Division of Translational Alzheimer Neurobiology, Karolinska Institutet, 141 57 Huddinge, Sweden; 2 MedTech West and the Department of Clinical Neuroscience and Rehabilitation, University of Gothenburg, 413 45 Gothenburg, Sweden; 3 Wolfson Molecular Imaging Centre, University of Manchester, Manchester, M20 3LJ, UK; 4 Department NVS, Center for Alzheimer Research, Division of Clinical Geriatrics, Karolinska Institutet, 141 57 Huddinge, Sweden; 5 Department of Geriatric Medicine, Karolinska University Hospital Huddinge, 141 86 Stockholm, Sweden; 6 Department of Psychology, Stockholm University, 106 91 Stockholm, Sweden; 7 Department NVS, Center for Alzheimer Research, Division of Neurogeriatrics, Karolinska Institutet, 141 57 Huddinge, Sweden; 8 Department of Surgical Sciences, Section of Nuclear Medicine & PET, Uppsala University, 751 85 Uppsala, Sweden; 9 Department of Chemistry, Uppsala University, 701 05 Uppsala, Sweden

## Abstract

Studying autosomal dominant Alzheimer’s disease (ADAD), caused by gene mutations yielding nearly complete penetrance and a distinct age of symptom onset, allows investigation of presymptomatic pathological processes that can identify a therapeutic window for disease-modifying therapies. Astrocyte activation may occur in presymptomatic Alzheimer’s disease (AD) because reactive astrocytes surround β-amyloid (Aβ) plaques in autopsy brain tissue. Positron emission tomography was performed to investigate fibrillar Aβ, astrocytosis and cerebral glucose metabolism with the radiotracers ^11^C-Pittsburgh compound-B (PIB), ^11^C-deuterium-L-deprenyl (DED) and ^18^F-fluorodeoxyglucose (FDG) respectively in presymptomatic and symptomatic ADAD participants (n = 21), patients with mild cognitive impairment (n = 11) and sporadic AD (n = 7). Multivariate analysis using the combined data from all radiotracers clearly separated the different groups along the first and second principal components according to increased PIB retention/decreased FDG uptake (component 1) and increased DED binding (component 2). Presymptomatic ADAD mutation carriers showed significantly higher PIB retention than non-carriers in all brain regions except the hippocampus. DED binding was highest in presymptomatic ADAD mutation carriers. This suggests that non-fibrillar Aβ or early stage plaque depostion might interact with inflammatory responses indicating astrocytosis as an early contributory driving force in AD pathology. The novelty of this finding will be investigated in longitudinal follow-up studies.

Alzheimer’s disease (AD) is a progressive brain disorder with gradually occurring cognitive decline. The time course of the underlying pathological changes remains largely veiled. Increasing evidence argues that these changes start decades before the onset of clinical symptoms. The order and magnitude of these processes are hitherto not well understood. The typical histopathology of AD includes the presence of β-amyloid (Aβ) plaques, neurofibrillary tangles, neuronal cell dysfunction and loss, and the activation of glial cells. It has been hypothesized that the accumulation of Aβ plaques plays a causative role in the disease development^1^. However, increasing evidence suggests that neurodegeneration might be triggered by a combination of processes, including tau deposition and neuroinflammation in addition to plaque accumulation^2^,^3^. The rapid development of cerebrospinal fluid (CSF) and positron emission tomography (PET) biomarkers has allowed modelling of the hypothetical sequence of biomarker changes in the presymptomatic, prodromal, and symptomatic stages of AD^4^,^5^.

AD is sporadic (sAD) in the great majority of cases, but 1–5% of Alzheimer patients suffer from the autosomal dominant form of the disease (ADAD), which is caused by mutations in the presenilin 1 (*PSEN1*), presenilin 2 (*PSEN2*), or amyloid precursor protein (*APP*) genes^6^. Within families harbouring a specific mutation, the age of onset for ADAD or early-onset familial AD (eoFAD) is predictable, which provides an opportunity for determining the sequence and magnitude of pathological changes that culminate in symptomatic disease^7^. Given the importance of understanding very early pathological changes in AD, members of families harbouring these mutations can be studied long before they develop any symptoms, providing an invaluable tool for research of these processes.

There are strong indications that factors other than Aβ, such as the activation of astroglia and microglia, and subsequent neuroinflammation, contribute to AD genesis and progression^8^,^9^,^10^. Most of our current understanding of astrocytosis stems from immunohistochemical studies in *postmortem* brain tissue^11^,^12^. Reactive astrocytes undergo structural and functional changes regulated by specific signalling events that occur in a context-dependent manner^13^. It has been observed that Aβ plaques are surrounded by activated astrocytes, and that activated astrocytes produce reactive oxygen and nitrogen species, which may contribute to AD pathogenesis. Nevertheless, much is still unknown regarding the relationship between reactive astrocytes and Aβ pathology^14^.

The PET tracer ^11^C-deuterium-L-deprenyl (DED) binds to monoamine oxidase B (MAO-B) on the outer mitochondrial membrane in astrocytes; increased DED binding is thought to reflect reactive astrocytosis^15^,^16^. In a ^11^C-DED PET study of sAD patients, we found evidence for early astrocytosis in ^11^C-PIB positive patients with mild cognitive impairment (MCI PIB+)^17^. DED binding was increased in MCI PIB+ patients compared to sAD patients and controls, suggesting that increased astrocytosis occurs in the earlier prodromal stages of AD^17^.

This cross-sectional study reports the baseline results from a large, ongoing, longitudinal study aiming to examine the temporal and regional relationships between astrocytosis, Aβ deposition, and glucose metabolism as a measure of neurodegeneration in ADAD and sAD. Here we demonstrate for the first time the presence of significant astrocytosis decades before the occurrence of clinical symptoms.

## Results

### Subjects

The demographic data for the subjects are presented in Table 1. There were significant differences in age, education, and mini-mental state examination (MMSE) scores between the groups. The presymptomatic mutation carriers in particular were considerably younger than members of the other groups. Differences in MMSE were anticipated due to the different clinical stages of the groups. The patients with MCI were subdivided into PIB positive (PIB+) and PIB negative (PIB−) subjects according to their global-to-cerebellum gray matter PIB retention ratios using a cut-off point of 1.41 derived from a larger multicenter study of PIB PET^18^.

### Neuropsychology

All raw test scores were transformed into z-scores (Table 1). Z-scores < −1.645 (fifth percentile) were considered outside the normal range. The sAD patients showed pathological z-scores in global cognition and episodic memory. Four of the eight MCI PIB+ patients had abnormal episodic memory compared to the population mean, and episodic memory had declined in the other four compared to previous assessments or they had abnormal values compared to their estimated premorbid function. One of the MCI PIB− patients showed abnormal episodic memory; the other two had abnormal performance in non-memory cognitive domains. The episodic memory z-scores were within the normal range for all non-carriers in ADAD families as well as for the six presymptomatic ADAD mutation carriers. Two of the three symptomatic ADAD carriers demonstrated results markedly outside the normal range in global cognition and episodic memory, while the third carrier had abnormal or close to abnormal results in two episodic memory tests. For detailed individual test scores, please refer to Table 2.

### Principal component analysis modeling and model quality

**Figure 1:**
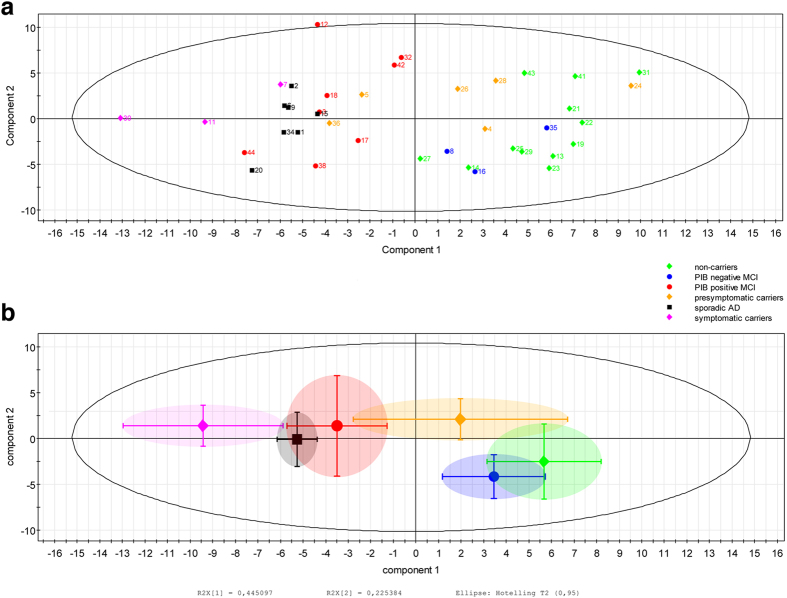
(**a**) Scatter plot displaying results from all examined individuals according to Principal Component Analysis (PCA). Distribution along the first two components is shown. (**b**) The figure displays a simplified summary of the PCA data for each group; the central shapes represent the mean PCA score for each group, bars represent standard deviations for each group on each principal component.

The principal component analysis PCA model accounted for 67% of the variance of the original data (R^2^(X)), and its cross-validated predictability, Q^2^(X), was 0.61 (considered a valid model)^19^. Figure 1a shows a scatter plot with the distribution of all participants’ data along two components. Figure 1b displays a simplified plot showing the means and standard deviations for each group. Supplementary Fig. S1 shows the influence of the 25 most important variables on each component. As demonstrated in Fig. 1a, the separation of the groups along component 1 shows a clear division between symptomatic ADAD mutation carriers, sAD patients, and MCI PIB+ patients on the one side and presymptomatic ADAD mutation carriers, MCI PIB− patients, and ADAD mutation non-carriers on the other. According to the loading plot, PIB retention and FDG uptake on the respective sides accounted for this separation (Fig. 1b; for detailed regional information, please see Supplementary Fig. S1). The order of rankings along the first component indicated highest PIB retention and lowest FDG metabolism for symptomatic ADAD mutation carriers, followed in order by sAD patients, MCI PIB+ patients, presymptomatic ADAD carriers, MCI PIB− patients, and ADAD non-carriers.

DED slope values were most important for separation along component 2. Here, although the pattern was not as clear as for component 1, the order of rankings suggested highest slope values for the presymptomatic ADAD mutation carriers, followed by MCI PIB+ patients, symptomatic ADAD carriers, sAD patients, ADAD non-carriers, and MCI PIB− patients (for detailed regional information, please see Supplementary Fig. S1).

### ^11^C-Pittsburgh compound-B PET region of interest analysis

**Figure 2:**
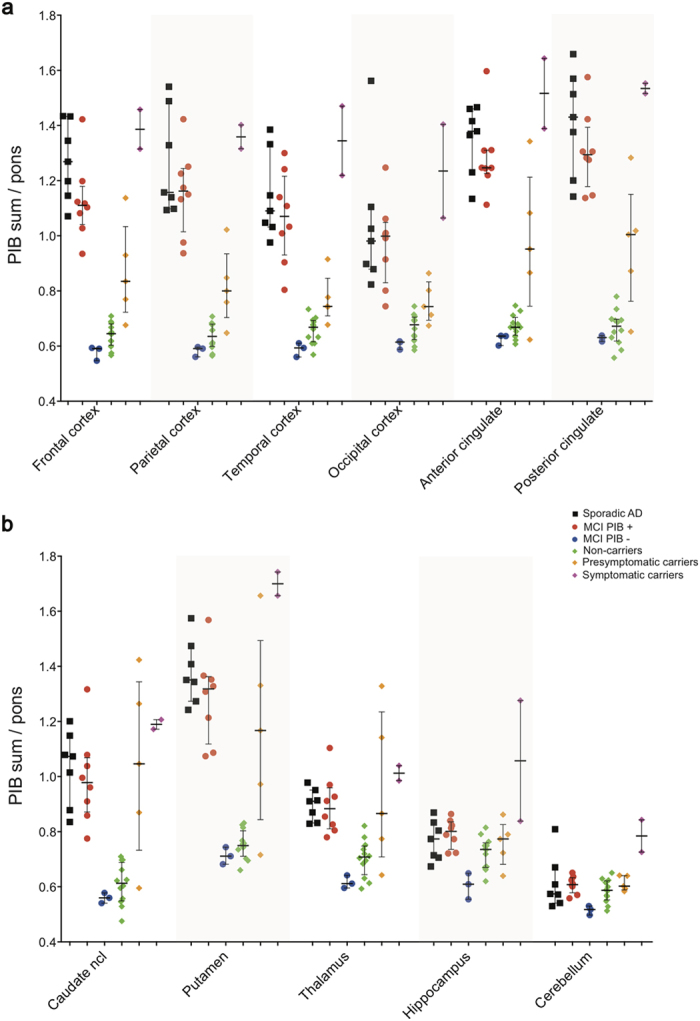
Scatter plots showing all individual PIB retention data in composite cortical (**a**) and subcortical (**b**) bilateral brain regions.

PIB retention differed significantly between the groups (Fig. 2, Supplementary Table S1a); Kruskal-Wallis tests in each of 11 ROIs for the four groups being compared (presymptomatic ADAD carriers, MCI PIB+, sAD and ADAD non-carriers), were significant (p < 0.001) in all ROIs except for the hippocampus. Highest PIB retention values were seen in the symptomatic mutation carriers in all brain regions, with particularly increased retention in the putamen (z-score = 17.3) and in the hippocampus (z-score = 9.7) of one carrier.

Following the highest PIB retention values observed in individual symptomatic carriers, the ranked order for PIB retention derived from Mann-Whitney pair-wise comparisons between groups and the z-scores for MCI PIB− patients was: sAD patients ≥ MCI PIB+ patients > presymptomatic ADAD mutation carriers > ADAD non-carriers ≥ MCI PIB−; this pattern is consistent with the results obtained from the PCA analysis. The ADAD non-carriers and the MCI PIB− subjects had persistently very low PIB retention scores in all the examined regions. Compared to ADAD non-carriers, the MCI PIB− patients had significantly lower PIB retention in the hippocampus (z-score = −2.1).

The sAD and MCI PIB+ patients had increased PIB retention compared to ADAD non-carriers with effect sizes r > 0.80 (p < 0.001) in all ROIs, except the hippocampus which did not show a significant difference. Both sAD and MCI PIB+ groups showed significantly increased PIB retention compared to presymptomatic ADAD mutation carriers in all cortical regions, most pronounced in parieto-temporal cortex (effect sizes r ~ 0.60–0.70, p < 0.05), but differences were not significant in subcortical regions. Presymptomatic mutation carriers had higher PIB than ADAD non-carriers in all ROIs (effect sizes r ~ 0.5–0.7, p < 0.05), except for the hippocampus.

### ^11^C-deuterium-L-deprenyl PET region of interest analysis

**Figure 3:**
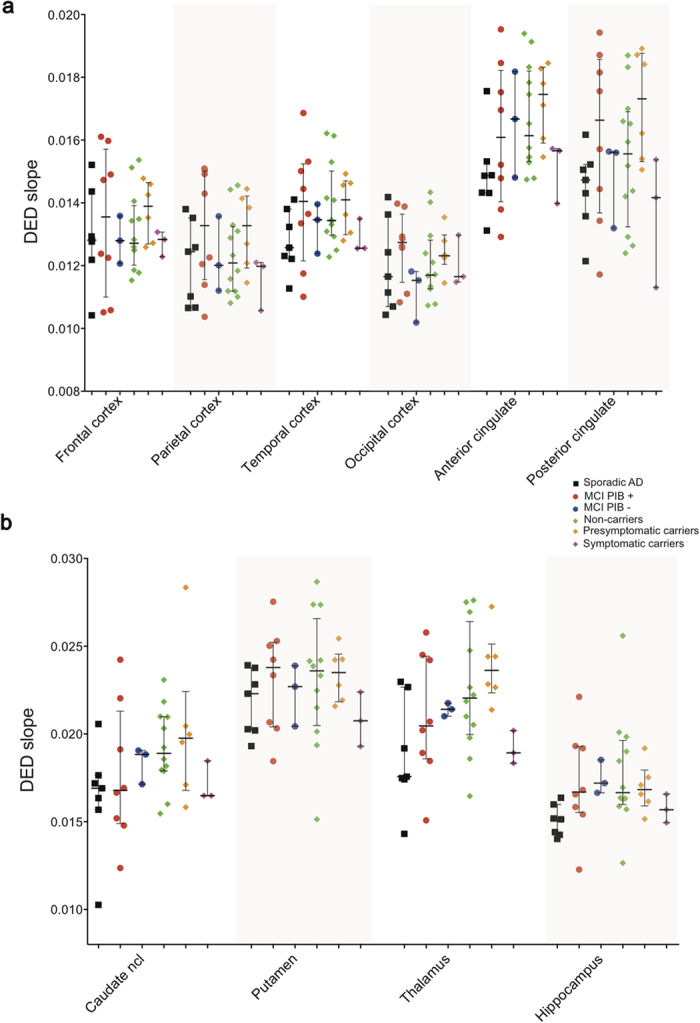
Scatter plots showing all individual DED slope data in composite cortical (**a**) and subcortical (**b**) bilateral brain regions. No data are shown for the cerebellum since this was used as the reference region in the modified Patlak reference tissue model.

Comparisons of four groups (presymptomatic ADAD carriers, MCI PIB+, sAD and ADAD non-carriers) using Kruskal-Wallis tests in each of 11 ROIs (except for the cerebellum), showed a trend toward statistical significance for the anterior cingulate cortex, the thalamus and the hippocampus (p = 0.059–0.069) (Supplementary Table S1b). The highest DED slope values were seen in the presymptomatic ADAD mutation carriers on average. The largest differences among groups were found between presymptomatic ADAD-carriers and sAD, where Mann-Whitney comparisons were significant in temporal, anterior/posterior cingulate, thalamus and hippocampus (r ~ 0.6–0.7, p < 0.05) (Fig. 3). The ADAD non-carrier group showed high variance, whereof two individuals (31 and 43 in Fig. 1a) showed very high DED slope values in most brain regions, which accounts for the rather high mean values in the non-carriers. Subject 43 showed particularly high values, especially in the hippocampus and the parahippocampal gyrus (not shown). sAD had lower DED than MCI PIB+ in the hippocampus (r = 0.51, p < 0.05), consistent with the results from the PCA analysis. The Mann-Whitney comparisons between sAD and ADAD non-carriers revealed significantly lower DED binding in sAD in anterior cingulate, caudate and hippocampus (r =0.48-0.56, p < 0.05).

### ^18^F-fluorodeoxyglucose PET region of interest analysis

**Figure 4:**
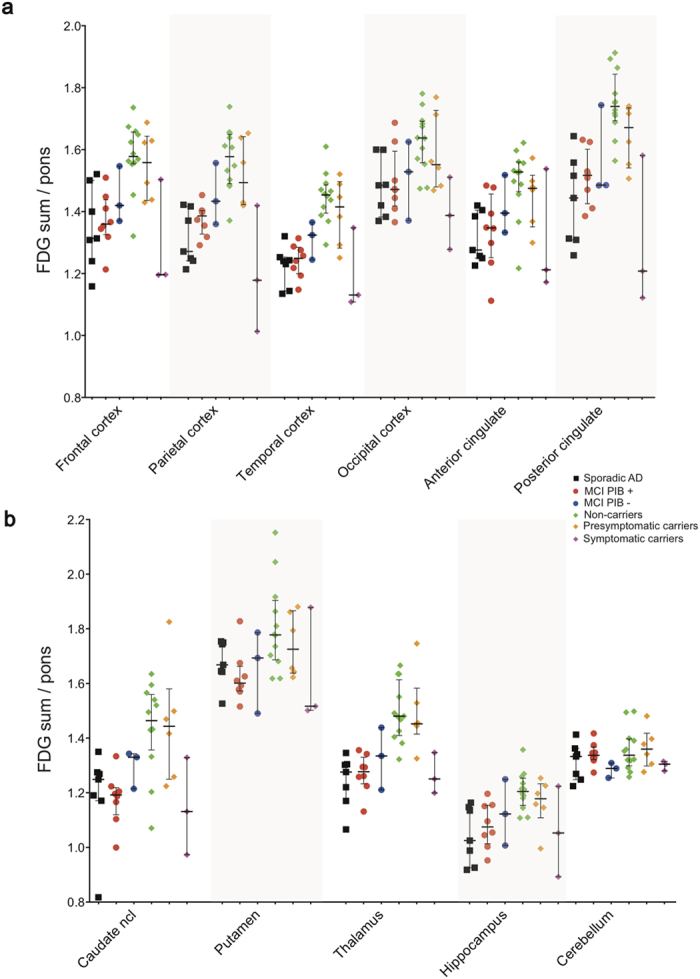
Scatter plots showing all individual FDG uptake data in composite cortical (**a**) and subcortical (**b**) bilateral brain regions.

The pattern of cerebral glucose metabolism as measured by FDG uptake was significantly different between the groups (Fig. 4, Supplementary Table S1c); the Kruskal-Wallis tests between the four groups being compared (presymptomatic ADAD carriers, MCI PIB+, sAD and ADAD non-carriers) showed significant results in all ROIs, most pronounced in parieto-temporal, posterior cingulate, and thalamus (p < 0.001). FDG uptake was lowest in individual symptomatic mutation carriers (most pronounced in parieto-temporal and posterior cingulate with z-scores between −3.2 and −4.2), followed by sAD and MCI PIB+ patients in almost all examined brain regions (Fig. 4). There were no significant differences in FDG uptake between sAD and MCI PIB+ patients. As expected, the highest FDG uptake occurred in the non-carriers, which was significantly higher (p < 0.05) than in sAD and in MCI PIB+ groups in all ROIs. The group of presymptomatic carriers showed to be statistically comparable to MCI PIB+ patients in anterior and posterior cingulate cortices as well as in the occipital region, indicating incipient hypometabolism in presymptomatic carriers. The pattern of glucose metabolism across groups was consistent with results from the PCA analysis.

### Statistical parametric mapping analysis

**Figure 5:**
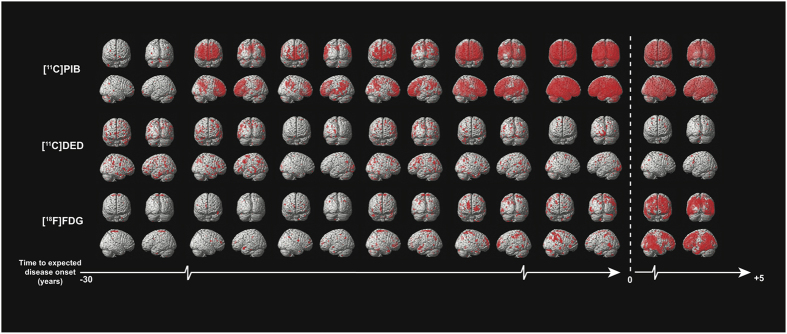
Progression of PET biomarkers in ADAD mutation carriers. Statistical parametric mapping (SPM) results are displayed for ADAD mutation carriers (*n* = 7). Each pair of columns represents an individual mutation carrier compared to five age-matched non-carriers. Each pair of rows represents a different PET biomarker. The scale from left to right represents the approximate time (in years) to the expected onset of clinical symptoms. All SPM clusters shown are significant at p < 0.001 (uncorrected). The figure demonstrates the progressive and heterogeneous nature of Alzheimer’s disease pathology as the expected onset of clinical symptoms approaches; neocortical fibrillar Aβ increases (PIB retention, top two rows), astrocytosis decreases (DED binding, middle two rows), glucose metabolism decreases (FDG hypometabolism shown, bottom two rows).

The statistical parametric mapping (SPM) results generally demonstrated ongoing progressive pathology in ADAD mutation carriers as the expected age at symptom onset approached (Fig. 5), consistent with the observed regional PET retention values in individual presymptomatic mutation carriers (Supplementary Table S2). The data demonstrated that PIB retention increases, DED binding decreases, and FDG uptake decreases close to and after the expected age of symptom onset. Increased DED binding was detectable at the earliest measured time point, nearly three decades before expected symptom onset. The pattern of changes for each PET tracer was not homogeneous for each individual.

## Discussion

The rapid development of molecular imaging has provided powerful tools for the detection of AD pathology at an early stage of the disease. These tools, in combination with the currently available biomarkers, provide an unprecedented opportunity to define a therapeutic window for use in the development of potential preventive/disease-modifying therapies. For example, high retention of specific Aβ PET tracers such as Pittsburgh compound-B in patients with mild cognitive impairment seems to predict a high risk of developing Alzheimer’s disease^18^,^20^, at a stage of the disease when cerebral glucose metabolism is less impaired. The new research diagnostic criteria for Alzheimer’s disease suggested by the International Working Group and the US National Institute on Aging–Alzheimer’s Association take into account the recent improved accessibility of CSF and imaging biomarkers^21^,^22^,^23^,^24^. Amyloid PET has been defined as a diagnostic marker that reflects *in vivo* pathology, while cerebral glucose hypometabolism is a downstream marker that monitors the course of neurodegeneration^23^. Studies in presymptomatic AD patients are important for further insight into the time course of these pathophysiological processes.

The amyloid cascade hypothesis, the focus of AD research for decades^1^, has received increasing scrutiny due to the recent unsuccessful treatment trials based on this line of thought^25^, and several other mechanisms including neuroinflammation have been suggested as promising alternative therapeutic targets^26^. Astrocyte activation has received increased attention due to its role in neuroinflammation^8^,^27^,^28^. However, astrocytosis is not completely understood; reactive astrocytes appear to have either neuroprotective or neurodegenerative effects at different stages of the evolution of the disease^8^,^29^.

The chronological order and relative causality of astrocyte activation, fibrillar Aβ deposition and glucose hypometabolism, have not been established, yet. Interestingly, it has been shown in cultured rat astrocytes that Aβ induces MAO-B expression^30^, which indicates a link between astrocyte activation and amyloid pathology. Astrocyte activation is a complex, regionally and temporally dependent phenomenon^31^, and MAO-B expression in reactive astrocytes differs across brain regions^16^. In addition, different markers of astrocytosis could detect different subpopulations of astrocytes and/or different stages in the disease. Reactive astrocytes have been measured in *postmortem* AD brain tissue using ^3^H-DED autoradiography and glial fibrillary acidic protein (GFAP) immunohistochemistry^31^,^32^,^33^. A strong regional correlation was observed between *postmortem* GFAP reactive astrocytes and both *in vivo*^11^C-PIB and *in vitro*^3^H-PIB binding. However, no correlation was found between *postmortem*^3^H-DED and *in vivo*^11^C-PIB^33^. There was no correlation either between ^3^H-DED reactive astrocytes and fibrillar Aβ in AD autopsy brains, using regional and laminar distribution analyses^31^. Evidence that neurodegenerative processes might not be solely dependent on Aβ plaque pathology is also based on *in vivo* PET imaging findings such as a lack of correlation between plaque deposition, metabolic dysfunction, atrophy, and clinical outcome (e.g. cognitive dysfunction) in AD patients^34^.

While there are several *in vitro* studies on reactive glia, less is known about their function *in vivo*. PET imaging of astrocytosis in mild cognitive impairment and sAD patients using ^11^C-DED has demonstrated elevated astrocytosis in MCI PIB+ patients (prodromal AD), suggesting that astrocytosis is an early event in sAD patients^17^. We have also recently reported that increased ^11^C-DED binding correlated with decreased gray matter density in the parahippocampus of MCI PIB+ patients^35^. However, no *in vivo* studies using ^11^C-DED in ADAD have been reported to date. Moreover, *in vivo* studies on astrocytosis are required to investigate the different roles of the reactive astrocytes across different disease stages, especially with regard to neuroprotection vs. neurotoxicity. Furthermore, microglia and astrocytes show differential relationships to Aβ pathology and seem to play different roles in inflammation in AD^14^,^36^. *In vivo* PET studies using tracers for the translocator protein (TSPO) as a marker for microglial activation, showed discordant results with both increased tracer uptake in AD patients as compared to healthy controls and no difference between patients and controls^37^,^38^,^39^,^40^. This discrepancy can partly be explained by a polymorphism of TSPO^41^. The time course and interrelationship of microglial and astrocytic involvement in AD-related inflammation are yet to be elucidated.

In this study, the finding of early astrocytosis roughly coinciding temporally but not necessarily spatially with fibrillar Aβ deposition is consistent with evidence from *postmortem* and *in vivo* studies suggesting a general lack of correlation between reactive astrocytes and fibrillar Aβ. The observed early astrocytosis might have been caused by Aβ oligomers or other pathological features such as intraneuronal hyperphosphorylated tau protein and adds support to the idea of glial activation as independent of other pathological substrates in AD.

The finding of early astrocytosis in the AD continuum is strengthened by recent findings of YKL-40, a potential astrocyte-derived biomarker measured in CSF, being strongly related to Aβ in AD patients and with tau pathology as a marker for neurodegeneration, most pronounced at early pre-dementia stages of AD^42^,^43^,^44^,^45^. Increased levels of CSF YKL-40 were furthermore related to cognitive decline and cortical thinning^43^,^46^.

We also compared cases of ADAD and sAD in this study, since a common neuropathological pathway has been suggested^47^, although the onset of symptoms occurs earlier and disease severity is greater in familial cases^48^. In recent cross-sectional^49^ and longitudinal^50^ ADAD studies, PIB, FDG, and atrophy were evaluated and compared in order to investigate regional and temporal differences in biomarker levels, in relation to the estimated years to symptom onset (EYO). PIB retention was increased in almost every cortical region, starting earlier than 15 EYO, in ADAD patients. Reduced FDG and cortical thinning were detected especially in the parietal and posterior cingulate cortex (PCC)/precuneus regions from 10 to 5 EYO. In our study in ADAD, MCI patients and sAD patients, we included assessment of ^11^C-DED binding in addition to PIB and FDG examinations. The PCA model showed a clear separation pattern between the different groups using the three different PET tracers in 24 brain regions allowed. Interestingly, the direction of the measured signal for the PET biomarkers PIB and FDG (which indicated worsening of amyloid deposition and glucose hypometabolism with time) differed from that for DED (which indicated decreased astrocytosis). This observation clearly reveals an opposite direction in time course between PIB and DED although these PET tracers show similar early presymptomatic presence but change differently in disease progression.

The highest level of DED binding was observed in presymptomatic ADAD mutation carriers, while DED binding was low in symptomatic ADAD mutation carriers. Interestingly, earlier studies in healthy subjects have reported an age-related increase in MAO-B activity in healthy human brains, as investigated *in vitro* at autopsy^16^ and *in vivo* using PET DED imaging^51^.

The increase in PIB retention occurred early in presymptomatic ADAD carriers, predominantly in the anterior and posterior cinguli and the basal ganglia. This pattern of early changes in the subcortical brain regions is in agreement with recent ADAD studies^50^, where high PIB retention was detected in regions such as the caudate nucleus and the pallidum, in the absence of atrophy. An early hypermetabolic phase 25 years before estimated symptom onset was detected in the precuneus and PCC, based on a linear model of FDG vs. EYO^50^. Furthermore, previous studies in ADAD patients have shown thalamic hypometabolism 20 years before estimated symptom onset^7^ and atrophy on average 6 years before symptom onset^52^ in asymptomatic *PSEN1* mutation carriers, suggesting that the thalamus may also be involved in early disease mechanisms in ADAD patients. Subtle cognitive dysfunction in ADAD patients has been detected relatively close to the onset of symptoms (from about five years EYO)^53^. However, our study observed that performance can be quite variable in subjects from five years before symptom onset, with some subjects showing greater impairment and others remaining cognitively normal.

To conclude, we have demonstrated that measures of astrocytosis in ADAD mutation carriers can be observed decades before symptom onset, possibly coinciding with early fibrillar Aβ plaque deposition, both of which are followed later on by impaired glucose metabolism. Multivariate analysis of the PET results clearly separated the subject groups in a different order for the PIB/FDG tracers compared to the DED tracer. The analysis also suggested that DED might not yield equally high levels of sensitivity or specificity as PIB/FDG. It appears, however, that astrocytosis is initiated very early on, possibly before or at a similarly early stage as fibrillar Aβ deposition; this supports the notion that non-fibrillar forms of amyloid might cause inflammatory responses but also supports the possibility that astrocytosis is an important early contributory driving force in AD pathology. The novelty and importance of these findings will be confirmed in ongoing longitudinal studies.

## Methods

### Participants

Forty-four participants were recruited from the Department of Geriatric Medicine, Karolinska University Hospital Huddinge, Stockholm, Sweden; four were excluded for missing data and one *PSEN1* carrier was excluded for incomplete penetrance of the mutation. Eleven MCI patients, seven sAD patients, and 21 ADAD family members were eligible for inclusion (demographic details are provided in Table 1).

The ADAD families are part of an ongoing longitudinal clinical study at the Karolinska Institute and were recruited without reference to their mutation status. The family members are regularly followed up clinically and examined using neuropsychological assessment, MRI, collection of CSF, and blood tests^54^. In this study, we included mutation carriers and non-carriers from families harbouring the Arctic *APP* (*APP*arc), Swedish *APP* (*APP*swe), and presenilin 1 (*PSEN1)* (p.H163Y) mutations. Among the 21 family members, nine were carrying an ADAD mutation: two carried the *APP*arc, two the *APP*swe, and five the *PSEN1* mutations. The presence of the mutations in the subjects was confirmed by sequencing.

All participants underwent complete clinical examination, DED-, PIB-, and FDG-PET, MRI, and neuropsychological testing. The PIB PET data from the two *APP*arc mutation carriers were not included in the analyses because, according to our previously published results, symptomatic carriers of this mutation do not show PIB PET retention, while exhibiting other clinical and biomarker features comparable with other mutation carriers^55^. The sAD subjects and one mutation carrier fulfilled the criteria for AD as outlined by the NINCDS-ADRDA^56^. The MCI patients and one symptomatic mutation carrier fulfilled the criteria for MCI as outlined by Petersen^57^. All subjects provided written informed consent to participate in the study, which was conducted according to the Declaration of Helsinki and subsequent revisions and was approved by the Regional Human Ethics Committee of Stockholm and the Isotope Committee of Uppsala University, Sweden.

### Neuropsychological evaluation

All participants underwent routine clinical neuropsychological testing, which involved tests for global cognitive function, language, visuospatial function, episodic memory, attention, and executive ability, typically within six months of the PET examinations. Test scores were converted into z-scores in comparison with a reference group of healthy elderly people from the Karolinska University Hospital Huddinge^58^, while controlling for demographic conditions. Table 1 presents the composite z-scores for global cognition (full-scale intelligence quotient; FSIQ) and episodic memory performance (average of three scores: Rey auditory verbal learning test, total learning and delayed retention test, and Rey Osterrieth retention test), while Table 2 provides detailed individual test results.

### PET image acquisition and processing

The PET investigations were performed at Uppsala PET center on ECAT EXACT HR+ (Siemens/CTI) and GE discovery ST PET/CT (GE Healthcare) scanners. The PET scans for all three radiotracers were commonly performed in the order PIB, DED, and FDG in each subject on the same day with 2–3 hours between tracer injections. For a few subjects, and due to tracer synthesis failures, two scans were within four weeks of the others. The orbito-meatal line was used to center the heads of the participants. The emission scans for the DED investigation consisted of 19 time frames (4 × 30 s, 8 × 60 s, 4 × 300 s and 3 × 600 s) with a total duration of 60 min; the emission scans for the PIB investigations consisted of 24 frames (4 × 30, 9 × 60, 3 × 180 and 8 × 300 s) over 60 min. A late 40–60 min PIB sum image was created and used for subsequent image analysis. For each FDG emission scan, seven frames (1 × 60 s, 1 × 1140 s, 5 × 300 s) were acquired over 45 min. A late 30–45 min FDG sum image was created and used for subsequent analysis. Patients were required to fast for 4 h preceding the FDG scan, which was performed in a quiet room with dimmed light and eyes closed. The mean injected doses for each tracer were DED: 209 ± 57 MBq, PIB: 217 ± 74 MBq, and FDG: 232 ± 45 MBq. All emission data were acquired in 3D mode and reconstructed with filtered back-projection using a 4 mm Hanning filter, resulting in a transaxial spatial resolution of 5 mm in the field of view. The matrix included 128 × 128 pixels, and a 2.5 zoom factor was used. The reconstructed frames were re-aligned to correct for patient motion during each PET scan. The PET protocol followed the protocol established in our previous publication^17^.

### MRI image acquisition

All patients and ADAD family members underwent structural T1 MPRAGE MRI scanning using a 3T (Siemens Trio) scanner at the Karolinska University Hospital Huddinge, Stockholm, on average within five months of the PET examinations.

### Region of interest PET image analysis

All images were processed and analysed using a probabilistic atlas approach, as described previously^17^. In short, all image analyses were performed in the space of the DED PET images to preserve the fidelity of these PET data. First, all DED data from 10–60 min for each participant was summed to create a DED PET sum image in native space. The individual T1 MR images were co-registered and re-sliced to their corresponding DED sum image (using SPM8; Functional Imaging Laboratory, Wellcome Department of Imaging Neuroscience, University College London). This step created a T1-weighted MR reference image for each participant in DED PET space. Subsequently, each patient’s PIB and FDG images were co-registered and re-sliced to their individual T1 MR reference image.

All T1 MR reference images were then segmented into gray and white matter tissue classes using SPM8 59. The resultant probabilistic gray matter map was thresholded at 0.5 to create a binary gray matter mask. An inverse non-linear transform parameter file was generated as part of the segmentation algorithm. The inverse parameter file allowed data in the MNI (Montreal Neurological Institute) space to be transformed back into a native DED PET image space. The inverse parameter file from each participant was used to transform a simplified digital probabilistic atlas^60^, consisting of 24 cortical and subcortical regions, into native DED PET space. These atlases were multiplied by the corresponding binary gray matter mask, which generated a specific gray matter digital atlas for each participant. This step resulted in a single digital atlas for each participant that could be easily applied to analyse all three sets of PET data without additional manipulation.

Raw co-registered and re-sliced FDG (Bq/ml) and PIB (Bq/ml) PET data for each patient were sampled using the same individual digital atlases. Using this method, mean FDG uptake and PIB retention values were measured for each atlas region, as described previously^17^. Regional gray matter ratio values were created for FDG and PIB by dividing by the respective mean uptake in the pons.

### ^11^C-deuterium-L-deprenyl PET data modelling

The PET data for each participant were analysed using the individual brain atlases generated in the steps described above. Regional parametric data were generated from dynamic DED data from 20 to 60 minutes. No arterial blood samples were available as an input function for the DED modelling because of the clinical character of the study. Instead, a modified Patlak reference tissue model was used for kinetic analysis, according to earlier study methods^17^,^61^. Cerebellar gray matter from the individual atlases was used as the reference region in this model. Because net tracer accumulation also occurred in the cerebellum, the model was modified by correcting *k*_*3*_ in the reference region for irreversible binding with a fixed correction factor of 0.01, which was the minimum value for correction still leading to linearization in the model. This graphical reference Patlak model resulted in two measurements: the intercept (initial tracer distribution volume) and the slope (k_I_ = net DED binding to MAO-B). Since our main interest was to evaluate DED binding we used the slope value in subsequent analyses.

### Principal component analysis

Principal component analysis (PCA)^62^ is a multivariate method implemented in the software package SIMCA-P+; Umetrics AB, Umea, Sweden^19^. It is an unsupervised method meaning it does not use *a priori* information about groups for the analysis. Statistically, PCA reduces the dimensionality and complexity of the data by finding lines and planes in the n-dimensional space (n = number of variables in the model) that approximates the data in the best way possible in the least squares sense. This gives us the opportunity to get an overview of the data to observe group belonging, trends and outliers. It is also possible to view relationships between the observations and the variables. One of the advantages of multivariate methods like PCA is that it can handle many more variables than observations^19^.

The PCA model was created to include all individuals: sAD, MCI PIB+ patients, MCI PIB− patients, ADAD mutation non-carriers, presymptomatic ADAD mutation carriers, and symptomatic ADAD mutation carriers. In total 72 variables (24 regions for PIB retention, FDG uptake, and DED binding slope) were included for each subject in the PCA analysis. The pre-processing steps mean-centering and unit variance scaling were performed. Mean-centering improves the interpretability of the data by subtracting the variable average, which repositions the data set around the origin. Large variance variables are more likely to be expressed in modelling than low variance variables. Consequently, unit variance scaling was selected to scale the data appropriately. This scaling method calculates the standard deviation of each variable. The inverse standard deviation is used as a scaling weight for each PET measurement. The results from the PCA were visualized by plotting the first two components in a scatter plot. Each point in the scatter plot represents one individual subject. Loading plots were also created to illustrate how the PET variables included in the model influenced the observed pattern in the scatter plot along each component (only the 25 most important variables are shown).

### Region of interest statistical analyses

For each PET tracer, regional mean uptake values were compared between groups using two-tailed non-parametric Kruskal-Wallis tests, followed by post-hoc Mann-Whitney U tests in SPSS software. Non-parametric tests were applied due to the small sample sizes. The groups with *n* ≥ 5 being compared were: presymptomatic ADAD mutation carriers, MCI PIB+, sporadic AD (sAD) and ADAD mutation non-carriers. The comparisons were performed for 11 bilateral ROIs: frontal, parietal, temporal, occipital, anterior and posterior cingulate cortices, caudate nucleus, putamen, thalamus, hippocampus and cerebellum. Significance level was set at p < 0.05. Size effects (r) of Mann-Whitney comparisons were calculated using r = z/(√N), where z is the Mann-Whitney z and N the sum of individuals from the two groups being compared. Due to the small sample sizes of the MCI PIB− and symptomatic ADAD mutation carrier groups, individual z-score values were obtained instead with reference to the ADAD non-carrier group; z-score values were considered abnormal at |z| > 1.645.

### Statistical parametric modeling analysis

The PET data (PIB, FDG, DED) from each participant, which had been realigned to the DED image in native space, were spatially normalized using non-linear transformation from the segmentation of the T1 MRI data. This resulted in PIB and FDG ratio (/pons) images and DED slope (binding; min^−1^) images in standard MNI space for each participant. After spatial normalization, each individual PET image from each ADAD mutation carrier was compared separately, using a two-sample t-test (SPM), with images from a group of five non-carriers (controls) who were the most proximal in age to the mutation carrier investigated. An explicit binary gray matter mask was used so that only voxels within the mask were compared in the SPM analysis. All SPM results for each tracer were analysed at a p value of <0.001 (uncorrected for multiple comparisons).

## Additional Information

**How to cite this article**: Schöll, M. *et al.* Early astrocytosis in autosomal dominant Alzheimer's disease measured *in vivo* by multi-tracer positron emission tomography. *Sci. Rep.* **5**, 16404; doi: 10.1038/srep16404 (2015).

## Supplementary Material

Supplementary Information

## Figures and Tables

**Table 1:** Demographic information.

	*Clinical patients*			*ADAD individuals*		
	sAD	MCI PIB+	MCI PIB−	Mutationnon-carriers	Presymptomaticmutationcarriers	Symptomaticmutationcarriers
*n*	7	8	3	12	6	3
Age (y)	64.1 ± 6.1^C,D^ (55–73)	61.9 ± 6.8^H^ (53–75)	64.3 ± 6.7^K^ (60–72)	54.3 ± 12.9^M^	43.5 ± 9.3	58.7 ± 5.0
Gender (m/f)	5/2	4/4	2/1	8/4	*	2/1
MMSE	24.4 ± 5.7^C,D,E^ (14–30)	27.7 ± 1.9 (24–30)	27.7 ± 2.3 (25–29)	29.3 ± 1.1 (27–30)	29.6 ± 0.6 (29–30)	16.0 ± 8.7 (11–26)
Education (y)	10.4 ± 1.8^A,B^ (8–13)	14.0 ± 2.5^G^ (11–18)	13.7 ± 2.1 (12–16)	11.3 ± 2.3	13.2 ± 2.6	10.0 ± 1.0
Mutations					PSEN1 H163Y; APPswe; APParc	PSEN1 H163Y; APPswe; APParc
Time to expected age at symptom onset (y)					−11.8 ± 8.1	1.9 ± 2.6
APOE ε	3/3: 2; 3/4: 2; 4/4: 3	3/3: 2; 3/4: 6	3/3: 2; 3/4: 1	2/3: 1; 3/3: 7; 3/4: 3; 4/4: 1	3/3: 3; 3/4: 3	3/3: 1; 2/4: 1; 3/4: 1
FSIQ global cognition composite z-score	−*2.3* ± *1.7*	−0.9 ± 1.8	−0.2 ± 1.4	−0.2 ± 1.5	0.3 ± 1.3	−*3.3* ± *1.9*
Episodic memory composite z-score^#^	−*2.3* ± *0.8*	−1.3 ± 0.7	−0.9 ± 0.9	−0.1 ± 0.5	0.0 ± 0.5	−*2.2* ± *1.1*
**	sAD	MCI PIB+	MCI PIB−	Non-carriers	Presymptomatic carriers	Symptomatic carriers
sAD		A	B	C	D	E
MCI PIB+	A		F	G	H	I
MCI PIB−	B	F		J	K	L
Non-carriers	C	G	J		M	N
Presymptomatic carriers	D	H	K	M		O
Symptomatic carriers	E	I	L	N	O	

All values are means ± SD (range), unless stated otherwise. Superscript letters indicate significant differences between groups (see legend ** for group comparison codes, Fisher’s LSD post-hoc test, p < 0.05). *Gender distribution of presymptomatic autosomal dominant AD mutation carriers is not revealed to preserve confidentiality; ^#^sum of Rey auditory verbal learning test, total learning and delayed retention test, and Rey Osterrieth retention test scores. sAD = sporadic Alzheimer’s disease; ADAD = autosomal dominant Alzheimer’s disease; APOE = apolipoprotein E; FSIQ = full-scale intelligence quotient; MCI = mild cognitive impairment; MMSE = mini-mental state examination; PIB = Pittsburgh compound-B. Z-scores below −1.645 were considered outside normal range (italics).

** Legend for pairwise comparisons using Fisher’s LSD post hoc test.

**Table 2:** Detailed neuropsychological test results.

	*Clinical patients*			*ADAD individuals*		
	sAD	MCI PIB+	MCI PIB−	Mutationnon-carriers	Presymptomaticmutation carriers	Symptomaticmutation carriers
WAIS-R Information	−*2.00 (2.23)*	−0.84 (0.60)	−0.89 (0.90)	−0.40 (1.03)	0.18 (0.53)	−0.75 (1.85)
WAIS-R Similarities	−1.52 (1.54)	−0.64 (1.18)	0.67 (0.79)	0.08 (1.11)	−0.15 (0.79)	−0.94 (0.94)
WAIS-R Block Design	−*2.23 (1.33)*	−0.85 (1.17)	0.23 (1.75)	0.43 (1.14)	0.73 (0.81)	−1.16 (1.42)
WAIS-R Digit Symbol	−1.17 (1.17)	−0.75 (1.07)	−0.19 (1.44)	0.20 (0.51)	−0.03 (0.36)	−1.56 (1.49)
Rey-Osterrieth copy	−*2.16 (3.79)*	−0.40 (0.54)	−0.30 (0.88)	−0.15 (0.46)	−0.28 (0.35)	−*3.55 (4.95)*
Rey-Osterrieth recall	−*2.43 (0.84)*	−1.37 (0.71)	−0.74 (1.16)	−0.59 (0.85)	−0.22 (0.53)	−1.61 (2.39)
RAVLT trial 1–5 total	−*1.89 (0.90)*	−1.30 (0.90)	0.62 (0.72)	−0.61 (0.67)	−0.50 (0.74)	−*2.13 (0.90)*
RAVLT delayed recall	−*1.88 (1.54)*	−*1.74 (0.60)*	−1.38 (0.66)	−0.38 (0.83)	−1.25 (1.07)	−*1.99 (1.01)*
Digit Span forward UD	−1.23 (0.73)	−0.75 (0.83)	−0.90 (1.29)	0.20 (1.07)	−0.18 (1.66)	−1.02 (1.05)
Corsi Span UD	−*2.20 (1.60)*	−*1.71 (1.70)*	−0.49 (2.93)	0.24 (1.20)	−0.93 (1.38)	−*1.90 (2.70)*
Trail Making A time (s)	−*3.01 (6.37)*	−1.14 (1.61)	−0.62 (0.65)	0.85 (0.60)	0.78 (0.47)	0.27 (0.08)
Trail Making B time (s)	−*1.69 (1.66)*	−0.80 (1.36)	−0.44 (1.01)	0.38 (0.42)	0.09 (0.36)	−*2.22 (2.92)*

All values are means (SD) of z-scores, z-scores below −1.645 were considered outside normal range (italics). WAIS-R: Wechsler Adult Intelligence Scale–Revised; RAVLT: Rey Auditory Verbal Learning Test.
