# Supplementary material for: Early astrocytosis in autosomal dominant Alzheimer’s disease measured in vivo by multi-tracer positron emission tomography
Source: Sci Rep. 2015 Nov 10;5:16404. doi: 10.1038/srep16404 (PMC4639762; doi:10.1038/srep16404)
Supplement: Supplementary Information [file srep16404-s1.pdf]

Supplementary Information for "Early astrocytosis in autosomal dominant Alzheimer's disease measured in vivo by multi-tracer positron emission tomography"

Michael Schöll,[1,2] Stephen F. Carter,[1,3] Eric Westman,[4] Elena Rodriguez-Vieitez,[1] Ove Almkvist,[1,5,6] Steinunn Thordardottir,[5,7] Anders Wall,[8] Caroline Graff,[5,7] Bengt Långström,[9] Agneta Nordberg[1,5]

[1] Department NVS, Center for Alzheimer Research, Division of Translational Alzheimer Neurobiology, Karolinska Institutet, 141 57 Huddinge, Sweden

[2] MedTech West and the Department of Clinical Neuroscience and Rehabilitation, University of Gothenburg, 413 45 Gothenburg, Sweden

[3] Wolfson Molecular Imaging Centre, University of Manchester, Manchester, M20 3LJ, UK

[4] Department NVS, Center for Alzheimer Research, Division of Clinical Geriatrics, Karolinska Institutet, 141 57 Huddinge, Sweden

[5] Department of Geriatric Medicine, Karolinska University Hospital Huddinge, 141 86 Stockholm, Sweden

[6] Department of Psychology, Stockholm University, 106 91 Stockholm, Sweden

[7] Department NVS, Center for Alzheimer Research, Division of Neurogeriatrics, Karolinska Institutet, 141 57 Huddinge, Sweden

[8] Department of Surgical Sciences, Section of Nuclear Medicine & PET, Uppsala University, 751 85 Uppsala, Sweden

[9] Department of Chemistry, Uppsala University, 701 05 Uppsala, Sweden

Corresponding author:

Agneta Nordberg, MD, PhD, professor, Karolinska Institutet, Dept NVS, Center for Alzheimer Research, Division of Translational Alzheimer Neurobiology. Karolinska University Hospital Huddinge, Novum 5th floor. 141 57 Huddinge, Sweden

E-mail: agneta.k.nordberg@ki.se. Phone: +46 8 585 854 67. Fax: +46 8 585 854 70.

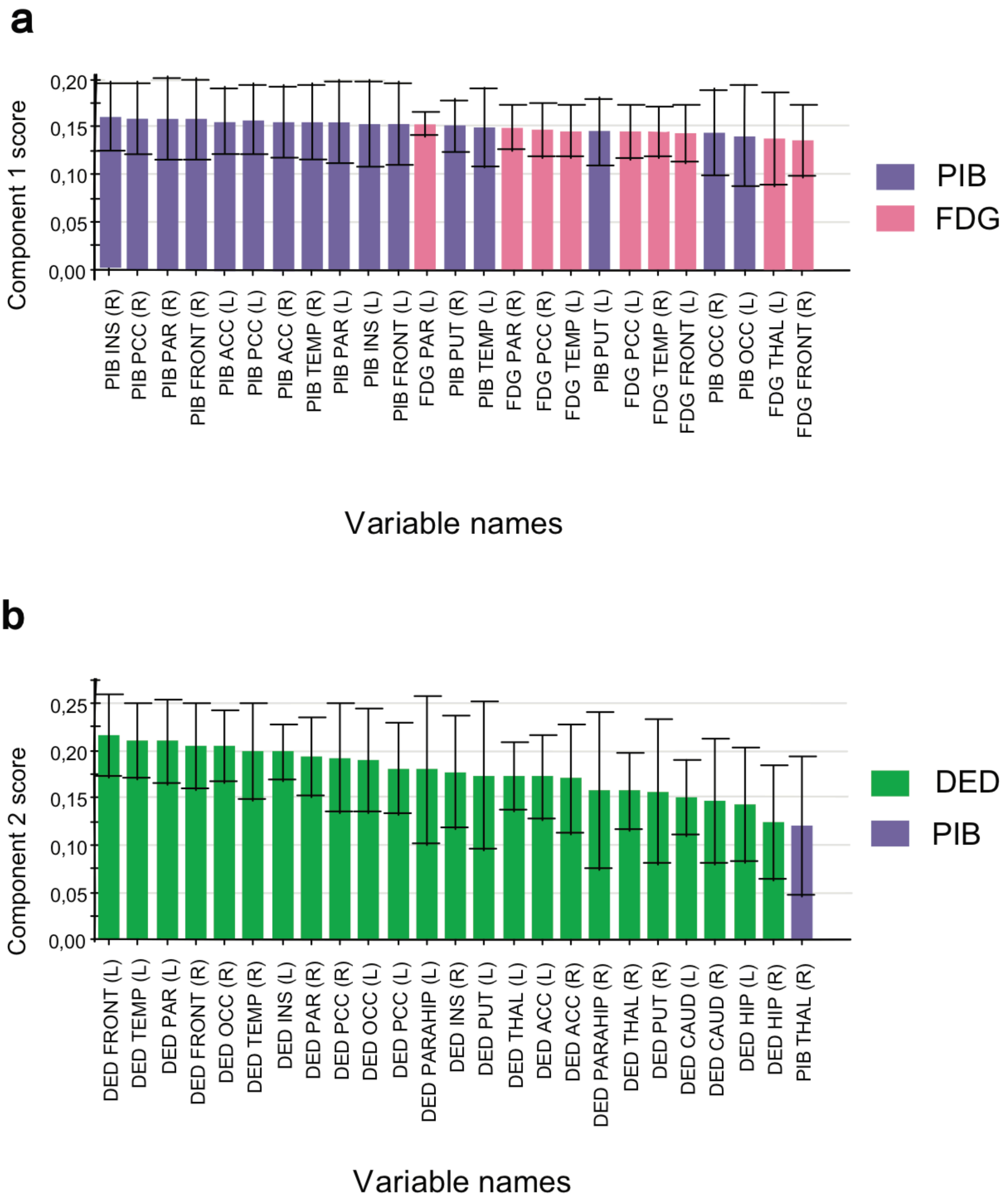

Supplementary Figure S1. (a,b) Loading plots visualizing the importance of each variable along the first two components of the Principal Components Analysis (PCA). Please observe that the importance value does not indicate increased or decreased PET values. L = left; R = right; ACC = anterior cingulate cortex; CAUD = caudate; FRONT = frontal; HIP = hippocampus; INS = insula; OCC = occipital cortex; PAR = parietal cortex; PARAHIPP = parahippocampus; PCC = posterior cingulate cortex; PUT = putamen; TEMP = temporal; THAL = thalamus.

Supplementary Table S1. Summary of group-level statistical tests for (a) PIB, (b) DED, and (c) FDG, using Kruskal-Wallis analysis of variance followed by pair-wise posthoc Mann-Whitney U test; all significant pair-wise comparisons included.

a

**Region of interest**	**PIB retention**<br>**Kruskal-Wallis test (p values)**	**PIB retention**<br>**Mann-Whitney U-test Effect size r (p value)**					
	**(sAD, MCI PIB+, pMC, NC)**	**pMC vs. NC**	**pMC vs. MCI PIB+**	**pMC vs. sAD**	**MCI PIB+ vs. NC**	**MCI PIB+ vs. sAD**	**sAD vs. NC**
**Cortical regions**							
Frontal	(p<0.001)***	r=0.69** (p=0.004)	r=0.57* (p=0.040)	r=0.61** (p=0.007)	r=0.83*** (p<0.001)	(p=0.064)	r=0.81*** (p<0.001)
Parietal	(p<0.001)***	r=0.61* (p=0.011)	r=0.73** (p=0.008)	r=0.65** (p=0.004)	r=0.83*** (p<0.001)	ns	r=0.81*** (p<0.001)
Temporal	(p<0.001)***	r=0.64** (p=0.008)	r=0.73** (p=0.008)	r=0.65** (p=0.004)	r=0.83*** (p<0.001)	ns	r=0.81*** (p<0.001)
Occipital	(p<0.001)***	r=0.54* (p=0.027)	r=0.65* (p=0.019)	r=0.61** (p=0.007)	r=0.81*** (p<0.001)	ns	r=0.81*** (p<0.001)
Anterior cingulate cortex	(p<0.001)***	r=0.51* (p=0.035)	(p=0.057)	r=0.58* (p=0.012)	r=0.83*** (p<0.001)	ns	r=0.81*** (p<0.001)
Posterior cingulate cortex	(p<0.001)***	r=0.56* (p=0.020)	r=0.69* (p=0.013)	r=0.58* (p=0.012)	r=0.83*** (p<0.001)	ns	r=0.81*** (p<0.001)
**Subcortical regions**							
Caudate	(p<0.001)***	r=0.59* (p=0.015)	ns	ns	r=0.83*** (p<0.001)	ns	r=0.81*** (p<0.001)
Putamen	(p<0.001)***	r=0.54* (p=0.027)	ns	ns	r=0.83*** (p<0.001)	ns	r=0.81*** (p<0.001)
Thalamus	(p<0.001)***	r=0.49** (p=0.045)	ns	ns	r=0.81*** (p<0.001)	ns	r=0.81*** (p<0.001)
Hippocampus	ns	ns	ns	ns	r=0.50* (p<0.025)	ns	ns

The Kruskal-Wallis test was performed to compare: presymptomatic mutation carriers (pMC), non-carriers (NC), MCI PIB+, and sAD patients. Presymptomatic carriers include those subjects with ages lower than the expected age at onset. Results of the Kruskal-Wallis test are reported as the significance value (p). The threshold for statistical significance was set at p = 0.05. Results of Mann-Whitney U-tests are reported in terms of effect size (r) and significance (p) for each pair of diagnostic groups; *p<0.05; **p<0.01; ***p<0.001.

b

Region of interest	DED binding<br><br>Kruskal-Wallis test (p values)	DED binding<br><br>Mann-Whitney U-test<br>Effect size r (p value)		
	**(sAD, MCI PIB+, pMC, NC)**	**pMC vs. sAD**	**MCI PIB+ vs. sAD**	**sAD vs. NC**
**Cortical regions**				
Frontal	ns	ns	ns	ns
Parietal	ns	(p=0.062)	ns	ns
Temporal	ns	r=0.59* (p=0.042)	ns	(p=0.091)
Occipital	ns	ns	ns	ns
Anterior cingulate cortex	(p=0.059)	r=0.73* (p=0.012)	ns	r=0.52* (p=0.022)
Posterior cingulate cortex	ns	r=0.68* (p=0.019)	ns	ns
**Subcortical regions**				
Caudate	ns	(p=0.062)	ns	r=0.48* (p=0.035)
Putamen	ns	(p=0.088)	ns	ns
Thalamus	(p=0.069)	r=0.68* (p=0.019)	ns	(p=0.076)
Hippocampus	(p=0.065)	r=0.59* (p=0.042)	r=0.51* (p=0.049)	r=0.56* (p=0.014)

The Kruskal-Wallis test was performed to compare: presymptomatic mutation carriers (pMC), non-carriers (NC), MCI PIB+, and sAD patients. Presymptomatic carriers include those subjects with ages lower than the expected age at onset. Results of the Kruskal-Wallis test are reported as the significance value (p). The threshold for statistical significance was set at p = 0.05. Results of Mann-Whitney U-tests are reported in terms of effect size (r) and significance (p) for each pair of diagnostic groups; *p<0.05; **p<0.01; ***p<0.001.

c

Regions of Interest	FDG uptake Kruskal-Wallis test (p values)	FDG uptake<br>Mann-Whitney U-test<br>Effect size r (p value)			
	(sAD, MCI PIB+, pMC, NC)	pMC vs. MCI PIB+	pMC vs. sAD	MCI PIB+ vs. NC	sAD vs. NC
**Cortical regions**					
Frontal	p=0.001**	r=0.69* (p=0.013)	r=0.63* (p=0.028)	r=0.67** (p=0.003)	r=0.72** (p=0.002)
Parietal	(p<0.001)***	r=0.73** (p=0.008)	r=0.82** (p=0.004)	r=0.74** (p=0.001)	r=0.76** (p=0.001)
Temporal	(p<0.001)***	r=0.73** (p=0.008)	r=0.77** (p=0.007)	r=0.79** (p<0.001)	r=0.79** (p=0.001)
Occipital	p=0.020*	ns	ns	r=0.53* (p=0.017)	r=0.58* (p=0.011)
Anterior cingulate cortex	p=0.006**	ns	r=0.68* (p=0.019)	r=0.59** (p=0.007)	r=0.62** (p=0.007)
Posterior cingulate cortex	(p<0.001)***	ns	r=0.63* (p=0.028)	r=0.78** (p=0.001)	r=0.78** (p=0.001)
**Subcortical regions**					
Caudate	p=0.002**	r=0.77** (p=0.005)	r=0.68* (p=0.019)	r=0.62** (p=0.005)	r=0.60** (p=0.009)
Putamen	p=0.020*	r=0.57* (p=0.040)	ns	r=0.62** (p=0.005)	(p=0.076)
Thalamus	(p<0.001)***	r=0.81** (p=0.003)	r=0.82** (p=0.004)	r=0.76** (p=0.001)	r=0.79** (p=0.001)
Hippocampus	p=0.002**	r=0.61* (p=0.028)	r=0.68* (p=0.019)	r=0.62** (p=0.005)	r=0.66** (p=0.004)

The Kruskal-Wallis test was performed to compare: presymptomatic mutation carriers (pMC), non-carriers (NC), MCI PIB+, and sAD patients. Presymptomatic carriers include those subjects with ages lower than the expected age at onset. Results of the Kruskal-Wallis test are reported as the significance value (p). The threshold for statistical significance was set at p = 0.05. Results of Mann-Whitney U-tests are reported in terms of effect size (r) and significance (p) for each pair of diagnostic groups; *p<0.05; **p<0.01; ***p<0.001.

Supplementary Table S2. Summary of PET retention data for individual presymptomatic carriers illustrated in Fig. 5, in temporal order (as measured by the estimated years to symptom onset) of increasing disease progression (left to right) for (a) PIB, (b) DED, and (c) FDG.

a

	PIB retention (SUVR/pons)					
**Region of interest**	**Mutation Carrier 1**	**Mutation Carrier 2**	**Mutation Carrier 3**	**Mutation Carrier 4**	**Mutation Carrier 5**	**Mutation Carrier 6**
**Cortical regions**						
Frontal	0.68	0.93	0.77	0.83	1.14	1.46
Parietal	0.65	0.85	0.76	0.80	1.02	1.40
Temporal	0.68	0.78	0.74	0.74	0.92	1.47
Occipital	0.71	0.74	0.67	0.80	0.86	1.40
Anterior cingulate cortex	0.62	1.08	0.87	0.95	1.34	1.65
Posterior cingulate cortex	0.65	1.00	0.87	1.02	1.28	1.55
**Subcortical regions**						
Caudate	0.60	1.26	1.05	0.87	1.42	1.21
Putamen	0.72	1.33	1.17	0.97	1.66	1.66
Thalamus	0.64	1.14	0.87	0.77	1.33	1.04
Hippocampus	0.64	0.79	0.72	0.77	0.86	1.28

b

	DED binding (min^{-1})					
**Region of interest**	**Mutation Carrier 1**	**Mutation Carrier 2**	**Mutation Carrier 3**	**Mutation Carrier 4**	**Mutation Carrier 5**	**Mutation Carrier 6**
**Cortical regions**						
Frontal	0.0143	0.0148	0.0126	0.0146	0.0135	0.0131
Parietal	0.0144	0.0138	0.0121	0.0141	0.0127	0.0121
Temporal	0.0146	0.0146	0.0128	0.0149	0.0136	0.0135
Occipital	0.0128	0.0123	0.0123	0.0135	0.0123	0.0130
Anterior cingulate cortex	0.0185	0.0183	0.0161	0.0171	0.0178	0.0157
Posterior cingulate cortex	0.0189	0.0184	0.0151	0.0187	0.0154	0.0153
**Subcortical regions**						
Caudate	0.0283	0.0195	0.0200	0.0205	0.0171	0.0165
Putamen	0.0254	0.0242	0.0228	0.0242	0.0220	0.0225
Thalamus	0.0272	0.0244	0.0213	0.0244	0.0228	0.0189
Hippocampus	0.0175	0.0192	0.0162	0.0171	0.0150	0.0156

c

	FDG uptake (SUVR/pons)					
**Region of interest**	**Mutation Carrier 1**	**Mutation Carrier 2**	**Mutation Carrier 3**	**Mutation Carrier 4**	**Mutation Carrier 5**	**Mutation Carrier 6**
**Cortical regions**						
Frontal	1.69	1.62	1.63	1.43	1.49	1.50
Parietal	1.65	1.64	1.56	1.43	1.42	1.42
Temporal	1.52	1.49	1.45	1.29	1.38	1.35
Occipital	1.71	1.77	1.54	1.56	1.48	1.51
Anterior cingulate cortex	1.57	1.48	1.50	1.30	1.47	1.54
Posterior cingulate cortex	1.72	1.73	1.74	1.63	1.51	1.58
**Subcortical regions**						
Caudate	1.83	1.41	1.50	1.26	1.47	1.33
Putamen	1.88	1.79	1.86	1.62	1.64	1.88
Thalamus	1.75	1.44	1.45	1.53	1.45	1.35
Hippocampus	1.25	1.23	1.16	1.14	1.19	1.23
